# Metabolic reprogramming: a new relevant pathway in adult adrenocortical tumors

**DOI:** 10.18632/oncotarget.5623

**Published:** 2015-11-16

**Authors:** Céline Pinheiro, Sara Granja, Adhemar Longatto-Filho, André M. Faria, Maria C. B. V. Fragoso, Silvana M. Lovisolo, Antonio M. Lerário, Madson Q. Almeida, Fátima Baltazar, Maria C. N. Zerbini

**Affiliations:** ^1^ Life and Health Sciences Research Institute (ICVS), School of Health Sciences, University of Minho, Braga, Portugal; ^2^ ICVS/3B's-PT Government Associate Laboratory, Braga/Guimarães, Portugal; ^3^ Barretos School of Health Sciences Dr. Paulo Prata – FACISB, Barretos, Sao Paulo, Brazil; ^4^ Molecular Oncology Research Center, Barretos Cancer Hospital, Barretos, Sao Paulo, Brazil; ^5^ Laboratory of Medical Investigation (LIM-14), Faculdade de Medicina da Universidade de São Paulo, São Paulo, Brazil; ^6^ Unidade de Suprarrenal, Disciplina de Endocrinologia e Metabologia, Laboratório de Hormônios e Genética Molecular LIM42, Hospital das Clínicas, Faculdade de Medicina da Universidade de São Paulo, São Paulo, Brazil; ^7^ Instituto do Câncer do Estado de São Paulo - ICESP, Hospital das Clínicas, Faculdade de Medicina da Universidade de São Paulo, São Paulo, Brazil; ^8^ Hospital Universitário, Faculdade de Medicina da Universidade de São Paulo, São Paulo, Brazil; ^9^ Departamento de Patologia, Faculdade de Medicina da Universidade de São Paulo, São Paulo, Brazil

**Keywords:** adrenocortical tumors, metabolic reprogramming, monocarboxylate transporter, Warburg effect

## Abstract

Adrenocortical carcinomas (ACCs) are complex neoplasias that may present unexpected clinical behavior, being imperative to identify new biological markers that can predict patient prognosis and provide new therapeutic options. The main aim of the present study was to evaluate the prognostic value of metabolism-related key proteins in adrenocortical carcinoma. The immunohistochemical expression of MCT1, MCT2, MCT4, CD147, CD44, GLUT1 and CAIX was evaluated in a series of 154 adult patients with adrenocortical neoplasia and associated with patients' clinicopathological parameters. A significant increase in was found for membranous expression of MCT4, GLUT1 and CAIX in carcinomas, when compared to adenomas. Importantly MCT1, GLUT1 and CAIX expressions were significantly associated with poor prognostic variables, including high nuclear grade, high mitotic index, advanced tumor staging, presence of metastasis, as well as shorter overall and disease free survival. In opposition, MCT2 membranous expression was associated with favorable prognostic parameters. Importantly, cytoplasmic expression of CD147 was identified as an independent predictor of longer overall survival and cytoplasmic expression of CAIX as an independent predictor of longer disease-free survival. We provide evidence for a metabolic reprogramming in adrenocortical malignant tumors towards the hyperglycolytic and acid-resistant phenotype, which was associated with poor prognosis.

## INTRODUCTION

Adrenocortical carcinoma (ACC) is a rare and highly aggressive malignancy with an annual incidence of 0.7– 2.0 cases per million [1–3]. Surgery is the standard of care for localized adrenocortical carcinomas. To date, systemic treatment of advanced ACC has shown unsatisfactory overall response [4, 5]. Over the past two decades, considerable advances have been made into the understanding of molecular mechanisms, but is still not yet satisfactory [1, 2, 6].

Adrenocortical neoplasms have been a great challenge since they may present an unexpected biological behavior when using classic features commonly used for histopathological classification of neoplastic lesions, particularly in the borderline Weiss (scores 3 and 4) group. Therefore, it is imperative to find alternative information that can help determine with greater accuracy the possible biological behavior of patients with this disease, and, consequently, establish a correct therapeutic strategy [7, 8].

During carcinogenesis and tumor progression, neoplastic cells may reprogram their metabolism, showing a preference for glycolysis pathway for energy production, even in the presence of oxygen, a phenomenon known as the “Warburg effect”. This aerobic glycolysis implies the conversion of pyruvate to lactic acid, leading to a reduction in intracellular pH [9]. To prevent acid-induced apoptosis as well as glycolysis inhibition by accumulation of the end product, cancer cells upregulate proteins related to pH regulation and lactate transport, including the glucose transporter GLUT1, the pH regulator carbonic anhydrase IX (CAIX) [10, 11] and monocarboxylate transporters (MCTs) [11]. Therefore, many malignancies show a significant increase in the expression of these plasma membrane transporters, with associations with poor patient's prognosis [12–15]. The MCT family has 14 members, being isoforms 1, 2 and 4 the most well studied isoforms responsible for the transport of monocarboxylates, including lactate, coupled with a proton across the plasma membrane. As consequence from their substrate affinities, MCT1 and MCT4 mediate monocarboxylate efflux from cells, while MCT2 is involved in monocarboxylate uptake [16]. Importantly, these transporters require co-expression with chaperones for proper plasma membrane localization and activity. The main chaperone of MCT1 and MCT4 is CD147 [17, 18], while MCT2 is mainly associated with gp70 [18]. CD44 has also been recently described as a MCT chaperone [19]; however CD147 and CD44 expressions do not account for all MCT1/4 positive cases, suggesting that an additional MCT chaperone still remains to be identified [20].

To the best of our knowledge, the metabolic profile of adrenocortical tumors is very little explored [21]. However, ^18^F-fluorodeoxyglucose positron emission tomography (FDG-PET) data suggest that adrenocortical carcinomas show high levels of glucose consumption [22–25], indicating a possible clinical relevance of the glycolytic metabolism for the management of this neoplasia. Given the importance of these neoplasms, particularly in the Brazilian environment [26, 27], and the potential use of molecular players of aerobic glycolysis as prognostic tools and therapeutic targets [10, 12, 28], the aim of the present work is to study the pattern of expression of the metabolism-related proteins MCT1, MCT2, MCT4, their chaperones CD147 and CD44, as well as GLUT1 and CAIX in adult ACC, and to determine whether these proteins have some biological and/or prognostic predictive value.

## RESULTS

### Expression of MCTs, CD147, CD44, GLUT1 and CAIX in adrenocortical adenomas and carcinomas

**Figure 1:**
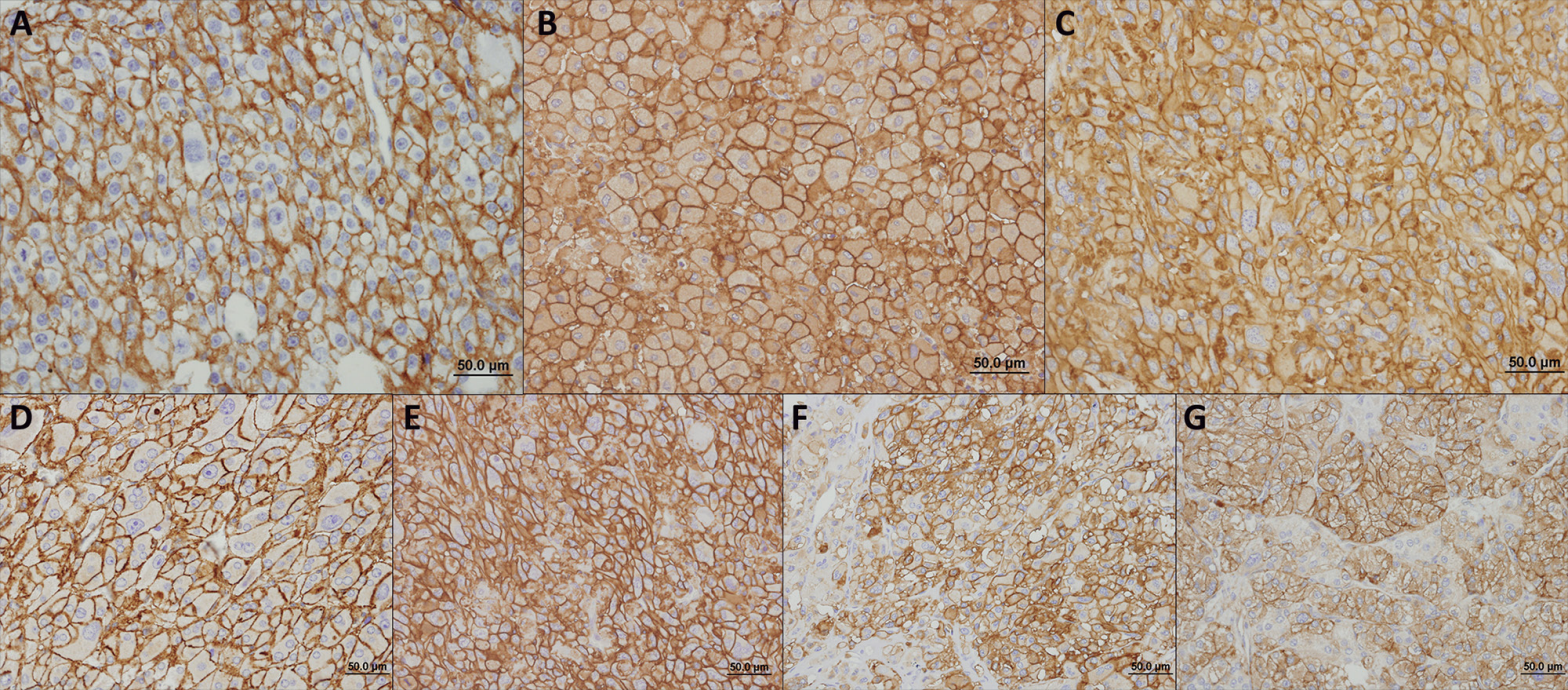
Immunohistochemical expression of MCT1 A. MCT2 B. MCT4 C. CD147 D. CD44 E. GLUT1 F. and CAIX G. in adrenocortical carcinomas All the proteins were more importantly found in the plasma membrane of cells.

**Figure 2:**
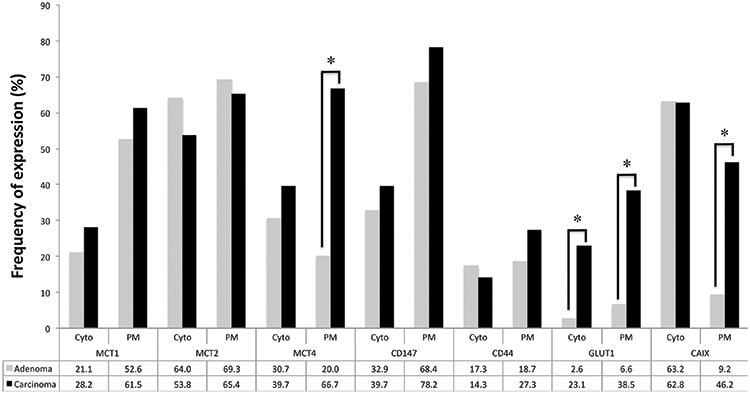
Frequency of staining of the different proteins analyzed in adrenocortical adenomas and carcinomas Pearson's chi-square (χ^2^) test was used to assess differences of expression frequency between adenomas and carcinomas. *p < 0.001

The immunohistochemical evaluation of these metabolism-related proteins in adrenocortical adenomas and carcinomas shows that all proteins can exhibit cytoplasmic, plasma membrane or simultaneous expression in both localizations, with a predominance of plasma membrane expression (exception for CAIX, Figures 1 and 2). As can be seen in Figure 2, all 3 MCT isoforms were expressed in the plasma membrane of most adrenocortical carcinoma samples (around 60–65%) and, MCT4 expression was significantly higher in carcinomas than in adenoma cells (66.7% *versus* 20.0%, respectively, *p* < 0.001). Cytoplasmic expression frequencies were more heterogeneous among MCT isoforms and did not differ between adenomas and carcinomas. CD147 was the protein more frequently expressed in the plasma membrane, but, similarly to CD44, no significant difference was observed between adenomas and carcinomas (Figure 2). In the carcinoma group, an increased expression was observed in the cytoplasm and plasma membrane for GLUT1 (*p* < 0.001 and *p* < 0.001, respectively, Figure 2), as well as in the plasma membrane for CAIX (*p* < 0.001, Figure 2). When evaluating the co-expression of MCTs with the other proteins at the plasma membrane (Table 1), we found co-expression of MCT1 and either CD147 (*p* = 0.001) or GLUT1 (*p* = 0.007), MCT2 co-expressed positively with CD147 (*p* < 0.001) and inversely with CAIX (*p* = 0.023), and MCT4 co-expressed with CD147 (*p* = 0.005), CD44 (*p* = 0.031), GLUT1 (*p* = 0.001) and CAIX (*p* < 0.001).

**Table 1:** Co-expression of MCTs with CD147, CD44, GLUT1 and CAIX, in adult adrenocortical tumor samples (adenomas and carcinomas). Only plasma membrane expressions were considered

	*CD147*			*CD44*			*GLUT1*			*CAIX*	
	*n*	Positive (%)	*p*	*n*	Positive (%)	*p*	*n*	Positive (%)	*p*	Positive (%)	*p*
**MCT1**			**0.001**			0.122			**0.007**		0.108
Negative	**66**	39 (59.1)		**65**	11 (16.9)		**66**	8 (12.1)		14 (21.2)	
Positive	**88**	74 (84.1)		**87**	24 (27.6)		**88**	27 (30.7)		29 (33.0)	
**MCT2**			**< 0.001**			0.150			0.293		**0.023**
Negative	**50**	26 (52.0)		**50**	8 (16.0)		**50**	14 (28.0)		20 (40.0)	
Positive	**103**	87 (84.5)		**102**	27 (26.5)		**103**	21 (20.4)		23 (22.3)	
**MCT4**			**0.005**			**0.031**			**0.001**		**< 0.001**
Negative	**86**	56 (65.1)		**85**	14 (16.5)		**86**	11 (12.8)		13 (15.1)	
Positive	**67**	57 (85.1)		**67**	21 (31.3)		**67**	24 (35.8)		30 (44.8)	

### Clinicopathological significance of the metabolism-related proteins

The analysis of MCT plasma membrane expression association with the clinicopathological parameters is shown in Table 2. MCT1 was significantly associated with stage III+IV (*p* = 0.029), MCT2 was significantly associated with smaller tumor size (*p* = 0.004), lower mitotic index (*p* = 0.008), absence of sinus invasion (*p* = 0.024) and absence of metastasis (*p* = 0.022), while MCT4 showed no significant associations with the clinicopathological data. Table 3 shows the associations of CD147, CD44, GLUT1 and CAIX plasma membrane expression with the clinicopathological parameters. CD147 was significantly associated with smaller tumor size (*p* = 0.009), GLUT1 was significantly associated with higher mitotic index (*p* = 0.011) and presence of metastasis (*p* = 0.004), CAIX was significantly associated with higher nuclear grade (*p* = 0.048) and presence of metastasis (*p* = 0.021), while CD44 showed no significant associations with the clinicopathological data. When evaluating the cytoplasmic expression of the proteins under study, MCT4 and CD147 were significantly associated with absence of metastasis and CAIX with absence of necrosis (data not shown).

**Table 2:** Association of plasma membrane expression of MCTs with the clinicopathological parameters in adult adrenocortical carcinomas

	*n*	*MCT1*		*MCT2*		*MCT4*	
		Positive (%)	*p*	Positive (%)	*p*	Positive (%)	*p*
**Tumour size**			0.111		**0.003**		0.536
< 8 cm	**18**	14 (77.8)		17 (94.4)		11 (61.1)	
≥8 cm	**58**	33 (56.9)		33 (56.9)		40 (69.0)	
**Tumour weight**			0.417		0.284		0.781
< 467.2 mg	**30**	18 (60.0)		21 (70.0)		21 (70.0)	
≥467.2 mg	**30**	21 (70.0)		17 (56.7)		20 (66.7)	
**Weiss score**			0.382		0.400		0.881
< 6	**33**	19 (57.6)		20 (60.6)		22 (66.7)	
≥6	**40**	27 (67.5)		28 (70.0)		26 (65.0)	
**Nuclear grade**			0.304		1.000		0.620
Low	**5**	2 (40.0)		4 (80.0)		3 (60.0)	
High	**36**	26 (72.2)		27 (75.0)		26 (72.2)	
**Mitotic index**			0.790		**0.008**		0.296
Low	**24**	16 (66.7)		22 (91.7)		15 (62.5)	
High	**17**	12 (70.6)		9 (52.9)		14 (82.4)	
**Atypical mitosis**			0.164		0.241		0.276
Absent	**28**	17 (60.7)		23 (82.1)		18 (64.3)	
Present	**13**	11 (84.6)		8 (61.5)		11 (84.6)	
**Tissue p53 *status***			0.118		1.000		0.272
Normal	**19**	10 (52.6)		10 (52.6)		12 (63.2)	
Mutated	**5**	5 (100.0)		3 (60.0)		5 (100.0)	
**Necrosis**			1.000		0.700		0.701
Absent	**11**	8 (72.7)		9 (81.8)		7 (63.6)	
Present	**30**	20 (66.7)		22 (73.3)		22 (73.3)	
**Venous invasion**			0.481		0.064		0.165
Absent	**27**	17 (63.0)		23 (85.2)		17 (63.0)	
Present	**14**	11 (78.6)		8 (57.1)		12 (85.7)	
**Sinus invasion**			0.645		**0.024**		1.000
Absent	**35**	23 (65.7)		29 (82.9)		25 (71.4)	
Present	**6**	5 (83.3)		2 (33.3)		4 (66.7)	
**Capsular invasion**			1.000		0.700		1.000
Absent	**27**	19 (70.4)		21 (77.8)		19 (70.4)	
Present	**13**	9 (69.2)		9 (69.2)		9 (69.2)	
**Staging**			**0.029**		0.186		0.674
I+II	**43**	22 (51.2)		31 (72.1)		28 (65.1)	
III+IV	**33**	25 (75.8)		19 (57.6)		23 (69.7)	
**Metastasis**			0.079		**0.022**		0.200
Absent	**37**	19 (51.4)		29 (78.4)		22 (59.5)	
Present	**41**	29 (70.7)		22 (53.7)		30 (73.2)	

**Table 3:** Association of plasma membrane expression of CD147, CD44, GLUT1 and CAIX with the clinicopathological parameters in adult adrenocortical carcinomas

	*CD147*			*CD44*			*GLUT1*			*CAIX*	
	*n*	Positive (%)	*p*	*n*	Positive (%)	*p*	*n*	Positive (%)	*p*	Positive (%)	*p*
**Tumour size**			**0.009**			0.238			0.299		0.075
<8 cm	**18**	18 (100.0)		**18**	7 (38.9)		**18**	5 (27.8)		5 (27.8)	
≥8 cm	**58**	42 (72.4)		**57**	14 (24.6)		**58**	24 (41.4)		30 (51.7)	
**Tumour weight**			0.347			0.937			0.592		1.000
<467.2 mg	**30**	25 (83.3)		**29**	8 (27.6)		**30**	10 (33.3)		14 (46.7)	
≥467.2 mg	**30**	22 (73.3)		**30**	8 (26.7)		**30**	12 (40.0)		14 (46.7)	
**Weiss score**			0.895			0.752			0.958		0.969
<6	**33**	26 (78.8)		**33**	8 (24.2)		**33**	13 (39.4)		15 (45.5)	
≥6	**40**	31 (77.5)		**40**	11 (27.5)		**40**	16 (40.0)		18 (45.0)	
**Nuclear grade**			1.000			0.645			0.382		**0.048**
Low	**5**	5 (100.0)		**5**	1 (20.0)		**5**	1 (20.0)		0 (0.0)	
High	**36**	35 (97.2)		**36**	13 (36.1)		**36**	16 (44.4)		20 (55.6)	
**Mitotic index**			1.000			0.591			**0.011**		0.279
Low	**24**	23 (95.8)		**24**	9 (37.5)		**24**	6 (25.0)		10 (41.7)	
High	**17**	17 (100.0)		**17**	5 (29.4)		**17**	11 (64.7)		10 (58.8)	
**Atypical mitosis**			1.000			0.734			0.075		0.265
Absent	**28**	27 (96.4)		**28**	9 (32.1)		**28**	9 (32.1)		12 (42.9)	
Present	**13**	13 (100.0)		**13**	5 (38.5)		**13**	8 (61.5)		8 (61.5)	
**Tissue p53 *status***			0.272			1.000			0.122		0.317
Normal	**19**	12 (63.2)		**19**	3 (15.8)		**19**	6 (31.6)		8 (42.1)	
Mutated	**5**	5 (100.0)		**5**	1 (20.0)		**5**	4 (80.0)		4 (80.0)	
**Necrosis**			0.268			0.140			0.736		0.335
Absent	**11**	10 (90.9)		**11**	6 (54.5)		**11**	4 (36.4)		4 (36.4)	
Present	**30**	30 (100.0)		**30**	8 (26.7)		**30**	13 (43.3)		16 (53.3)	
**Venous invasion**			1.000			1.000			0.424		0.153
Absent	**27**	26 (96.3)		**27**	9 (33.3)		**27**	10 (37.0)		11 (40.7)	
Present	**14**	14 (100.0)		**14**	5 (35.7)		**14**	7 (50.0)		9 (64.3)	
**Sinus invasion**			1.000			0.645			0.066		1.000
Absent	**35**	34 (97.1)		**35**	13 (37.1)		**35**	12 (34.3)		17 (48.6)	
Present	**6**	6 (100.0)		**6**	1 (16.7)		**6**	5 (83.3)		3 (50.0)	
**Capsular invasion**			1.000			1.000			0.581		0.311
Absent	**27**	26 (96.3)		**27**	9 (33.3)		**27**	10 (37.0)		12 (44.4)	
Present	**13**	13 (100.0)		**13**	5 (38.5)		**13**	6 (46.2)		8 (61.5)	
**Staging**			0.550			0.521			0.251		0.193
I+II	**43**	35 (81.4)		**42**	13 (31.0)		**43**	14 (32.6)		17 (39.5)	
III+IV	**33**	25 (75.8)		**33**	8 (24.2)		**33**	15 (45.5)		18 (54.5)	
**Metastasis**			0.559			0.263			**0.004**		**0.021**
Absent	**37**	30 (81.1)		**36**	12 (33.3)		**37**	8 (21.6)		12 (32.4)	
Present	**41**	31 (75.6)		**41**	9 (22.0)		**41**	22 (53.7)		24 (58.5)	

### Survival analysis

**Figure 3:**
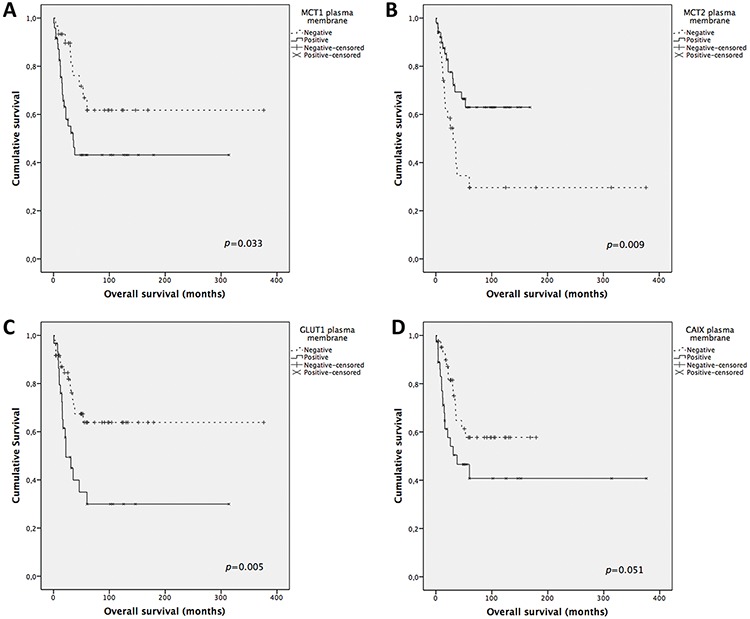
Overall survival curves of adrenocortical carcinomas' patients The results are stratified according to protein immunohistochemical expression. Only significant (or borderline) results are shown. Continuous line refers to positive expression while interrupted line refers to negative expression. **A.** Plasma membrane expression of MCT1; **B.** Plasma membrane expression of MCT2; **C.** Plasma membrane expression of GLUT1; **D.** Plasma membrane expression of CAIX.

**Figure 4:**
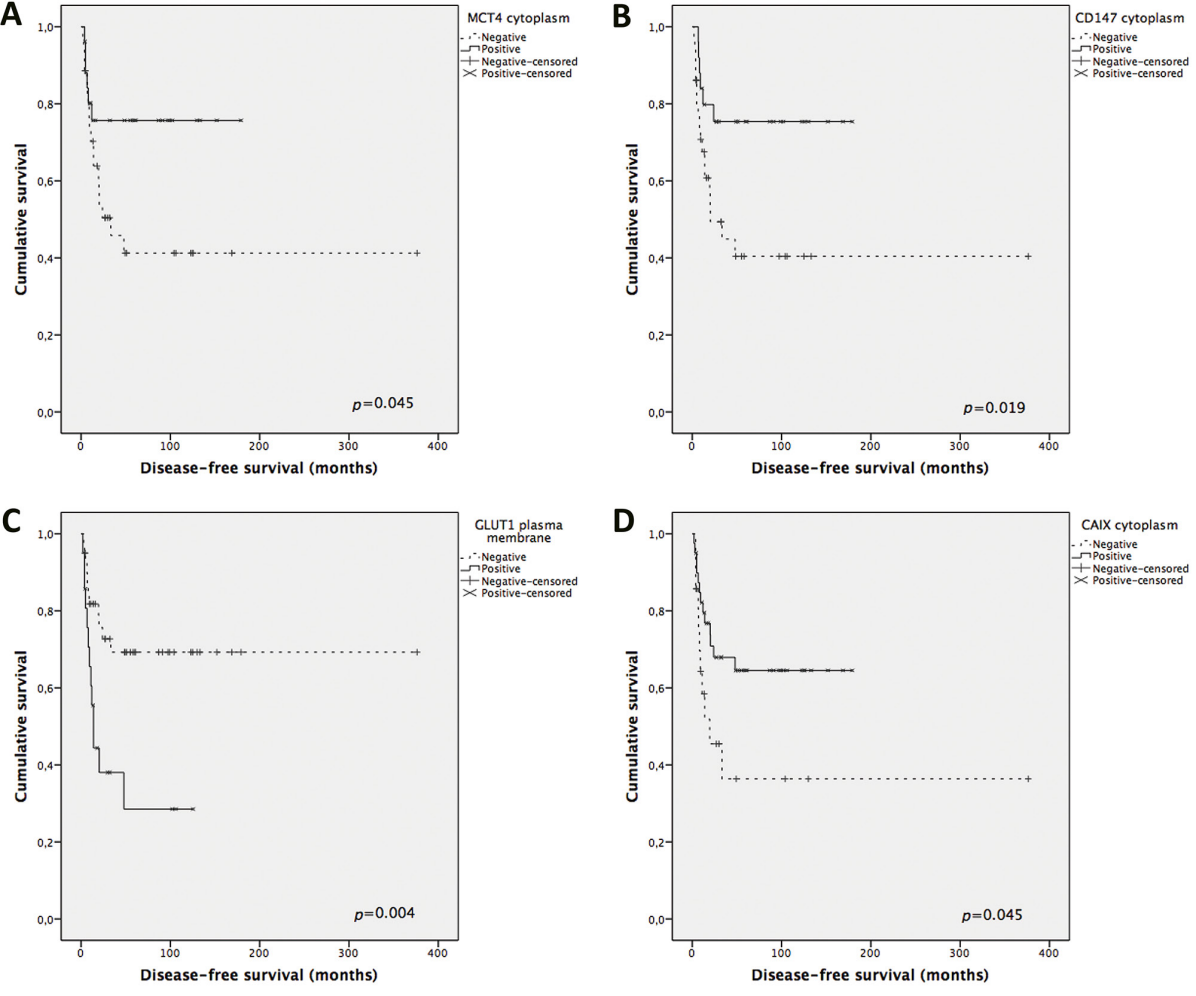
Disease-free survival curves of adrenocortical carcinomas' patients The results are stratified according to protein immunohistochemical expression. Only significant results are shown. Continuous line refers to positive expression while interrupted line refers to negative expression. **A.** Cytoplasmic expression of MCT4; **B.** Cytoplasmic expression of CD147; **C.** Plasma membrane expression of GLUT1; **D.** Cytoplasmic expression of CAIX.

Overall survival analysis (Figure 3) shows MCT1 and GLUT1 plasma membrane expression significantly associated with shorter overall survival (*p* = 0.033 and *p* = 0.005, respectively), MCT2 plasma membrane expression significantly associated with longer overall survival (*p* = 0.009), a tendency for CD147 cytoplasmic expression to be associated with longer overall survival (*p* = 0.058, data not shown) and CAIX plasma membrane expression associated with shorter overall survival (*p* = 0.051). Disease-free survival analysis (Figure 4) shows MCT4, CD147 and CAIX cytoplasmic expression to be significantly associated with longer disease-free survival (*p* = 0.045, *p* = 0.019 and *p* = 0.045, respectively), GLUT1 plasma membrane expression associated with shorter disease-free survival (*p* = 0.007) and a tendency for CAIX plasma membrane expression to be associated with shorter disease-free survival (*p* = 0.064, data not shown). The predictive prognostic values of the proteins were analyzed by means of Cox proportional hazards regression models (Table 4). Univariate analysis showed similar results to the ones obtained with Kaplan-Meier analysis. Multivariate analysis showed that CD147 cytoplasmic expression was the only factor with predictive value for overall survival, with a hazard ratio of 0.32 (*p* = 0.008), and that CAIX cytoplasmic expression was the only factor with predictive value for disease-free survival, with a hazard ratio of 0.31 (*p* = 0.036). Tumor stage according to ENSAT system confirmed to be a strong prognostic factor for both overall survival and disease-free survival in the univariate and multivariate analysis.

**Table 4:** Prognostic factors for overall survival and disease-free survival in adult adrenocortical carcinomas

	*Univariate analysis*			*Multivariate analysis*		
	Overall survival					
	HR	95% CI	*p*	HR	95% CI	*p*
**Male sex**	0.73	0.28–1.87	0.51			
**Age (yrs)**	0.99	0.98–1.02	0.76			
**Tumor size ≥ 8 cm**	3.86	1.37–10.9	**0.01**	3.6	0.7–17.5	0.13
**Weiss score ≥ 6**	0.86	0.44–1.67	0.64			
**Tumor stage (ENSAT 3)**	5.1	2.4–10.8	**0.0001**	2.3	1.5–8.8	**0.005**
**MCT1 cytoplasm**	1.05	0.47–2.3	0.91			
**MCT1 plasma membrane**	2.27	1.04–4.91	**0.038**	2.3	0.91–5.7	0.077
**MCT2 cytoplasm**	0.83	0.41–1.67	0.6			
**MCT2 plasma membrane**	0.409	0.20–0.82	**0.01**	0.78	0.34–0.74	0.548
**MCT4 cytoplasm**	0.53	0.24–1.14	0.1			
**MCT4 plasma membrane**	1.59	0.71–3.55	0.26			
**CD147 cytoplasm**	0.49	0.232–1.04	0.063	0.32	0.14–0.74	**0.008**
**CD147 plasma membrane**	0.94	0.44–2.05	0.89			
**CD44 cytoplasm**	0.63	0.2–1.8	0.39			
**CD44 plasma membrane**	0.9	0.4–2.0	0.79			
**GLUT1 cytoplasm**	1.3	0.6–2.81	0.5			
**GLUT1 plasma membrane**	2.63	1.3–5.3	**0.007**	1.97	0.9–4.2	0.088
**CAIX cytoplasm**	0.545	0.27–1.1	0.092	0.72	0.32–1.62	0.43
**CAIX plasma membrane**	1.98	0.98–3.99	0.056	0.96	0.42–2.18	0.93
						
	**Disease-free survival**					
	**HR**	**95% CI**	***p***	**HR**	**95% CI**	***P***
**Male sex**	1.1	0.45–2.8	0.81			
**Age (yrs)**	1.06	0.98–1.03	0.63			
**Tumor size 8 ≥ cm**	3.49	1.2–10.4	**0.022**	1.76	0.45–6.8	0.41
**Weiss score ≥ 6**	0.695	0.33–1.46	0.33			
**Tumor stage (ENSAT 3)**	4.46	2.1–9.5	**0.0001**	3.96	1.4–10.9	**0.008**
**MCT1 cytoplasm**	1.44	0.58–3.53	0.43			
**MCT1 plasma membrane**	2.1	0.84–5.1	0.11			
**MCT2 cytoplasm**	1.32	0.56–3.1	0.52			
**MCT2 plasma membrane**	0.42	0.18–0.97	**0.04**	0.74	0.28–1.9	0.527
**MCT4 cytoplasm**	0.42	0.16–1.08	0.071	1.12	0.36–3.5	0.843
**MCT4 plasma membrane**	1.22	0.497–2.99	0.66			
**CD147 cytoplasm**	0.36	0.14–0.92	**0.033**	0.43	0.16–1.18	0.1
**CD147 plasma membrane**	1.02	0.37–2.76	0.97			
**CD44 cytoplasm**	0.23	0.03–1.85	0.17			
**CD44 plasma membrane**	0.99	0.35–2.77	0.98			
**GLUT1 cytoplasm**	0.55	0.18–1.61	0.27			
**GLUT1 plasma membrane**	3.84	1.52–9.68	**0.004**	2.4	0.86–6.74	0.097
**CAIX cytoplasm**	0.47	0.2–1.1	0.079	0.31	0.1–0.92	**0.036**
**CAIX plasma membrane**	1.95	0.84–4.53	0.12			

### Glycolytic *versus* oxidative phenotype

**Figure 5:**
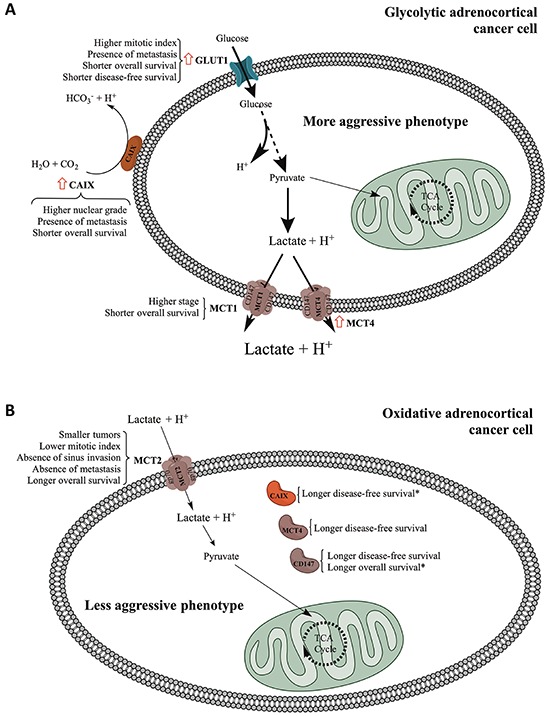
Schematic representation summarizing the major findings of the present work The schematic representation distinguishes the major findings for plasma membrane and cytoplasmic expression of the different proteins studied. Red arrow: significant increase in expression frequency when comparing adenomas to carcinomas. * Independent predictors of survival as determined by multivariate analysis.

**Figure 6:**
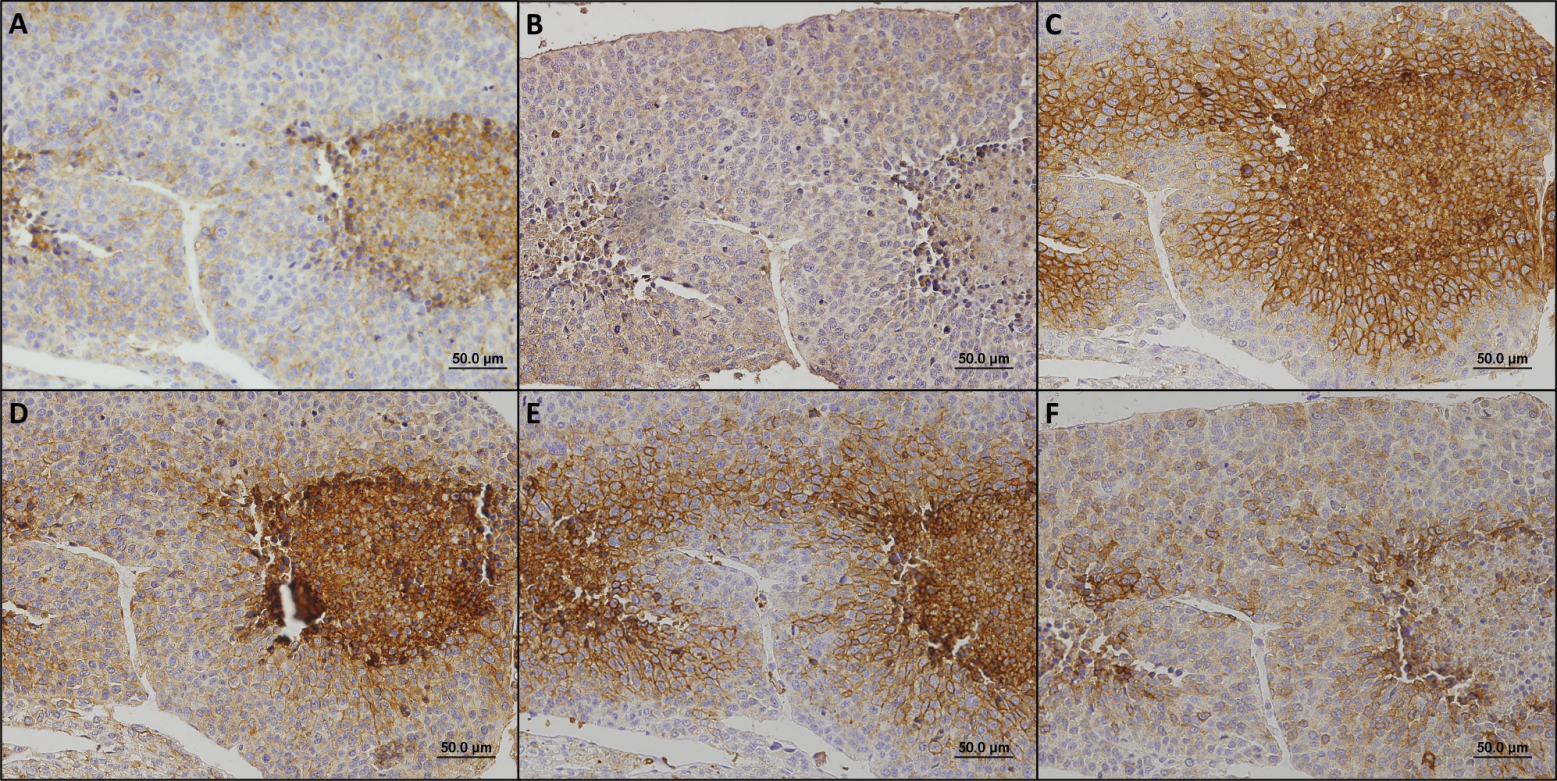
Immunohistochemical staining patterns of metabolism-related proteins in a case representative of the glycolytic phenotype In this case, MCT1 **A.** MCT4 **C.** CD147 **D.** GLUT1 **E.** and CAIX **F.** were found in the plasma membrane while MCT2 **B.** was observed in the cytoplasm.

**Figure 7:**
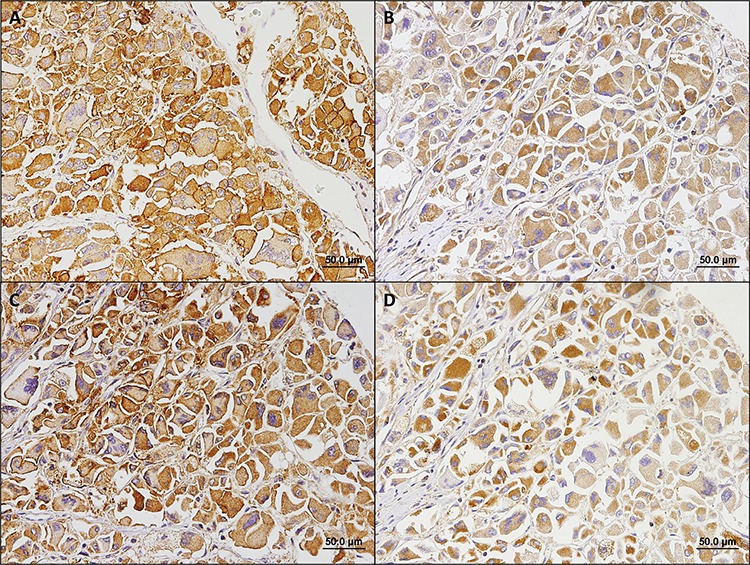
Immunohistochemical staining patterns of metabolism-related proteins in a case representative of the oxidative phenotype In this case, MCT2 **A.** was found in the plasma membrane, while MCT4 **B.** CD147 **C.** and CAIX **D.** were found in the cytoplasm.

The major findings described so far allowed us to establish 2 predominant expression profiles, the glycolytic and the oxidative phenotypes (summarized in Figure 5), which were associated with the clinicopathological parameters. The staining patterns of the metabolism-related proteins in cases representative of the glycolytic and oxidative phenotypes are shown in Figure 6 and Figure 7, respectively. The glycolytic phenotype was significantly associated with the mitotic index of the cases [8.3% (2/24) of cases with low mitotic index show a glycolytic phenotype *versus* 35.3% (6/17) of cases with high mitotic index; *p* = 0.049] and tumor p53 *status* [10.5% (2/19) of cases with normal p53 show a glycolytic phenotype *versus* 80.0% (4/5) of cases with mutated p53; *p* = 0.006].

## DISCUSSION

The increased frequency of MCT4, GLUT1 and CAIX plasma membrane expression of in adrenal cortical carcinomas suggests a metabolic remodeling of malignant cells towards a hyperglycolytic and acid-resistant phenotype, which is compatible with data from FDG-PET [22–25]. MCT2, mainly responsible for the uptake of monocarboxylates into cells [16], as expected, showed a slight but not significant decrease in the frequency of expression from adenomas to carcinomas, which is compatible with the metabolic reprogramming towards aerobic glycolysis in carcinomas. Although CD147 and CD44 showed no increase in expression in carcinomas, they were more frequently expressed in MCT positive tumors, which is in accordance with their role as MCT chaperones [17–19]. Notably, CD147 and CD44 did not account for all MCT1 or MCT4 positive tumors, suggesting the existence of an additional MCT chaperone, as already advocated by other study [20].

As vastly described, the metabolic reprograming of cancer cells is also involved in cancer cell aggressiveness and therapeutic resistance, rendering patients a poor prognosis [10]. The prognostic value of the metabolism-related proteins evaluated in the present study has been studied in a variety of tumor types; however, only GLUT1 has been evaluated in adrenocortical carcinoma [21]. In Fenske and colleagues study, GLUT1 was expressed in 33% of adrenocortical carcinoma samples, which is similar to the expression frequency found in the present study [21]. Herein, we show associations between GLUT1 and high mitotic index, presence of metastasis, and both longer overall and disease-free survival. This evidence is in agreement with the crucial role of GLUT1 in the metabolic reprogramming, as cancer cells are only able to depend on glycolytic pathway for energy production if the glycolytic flow is highly increased. This demands an increased uptake of glucose, mainly provided by GLUT1 in cancer cells [29].

MCT1 expression at the plasma membrane suggests a high lactate efflux from cancer cells, which, besides allowing glycolytic flux for energy production, also detains an important role in the microenvironment, by decreasing the immune response against tumor cells [30], increasing tumor cell motility [31], inducing angiogenesis [32], as well as stimulating hyaluronan and its receptor CD44, molecules involved in the process of cancer invasion and metastasis [33]. These roles of lactate in the extracellular milieu are in accordance with the association of MCT1 expression with high stage and shorter overall survival. In opposition, plasma membrane expression of MCT4, which was increased from adenomas to carcinomas, showed no association with the clinicopathological parameters. However, cytoplasmic expression of MCT4, as well as CD147, was associated with absence of metastasis. In accordance, cytoplasmic expression of MCT4 and CD147 was also associated with longer disease-free survival, while cytoplasmic expression of CD147 also showed a tendency to be associated with longer overall survival. Further studies are warranted to elucidate the role of MCT4 in the cytoplasm. Importantly, MCT2 plasma membrane expression was associated with good prognostic variables, including small tumor size, low mitotic index, absence of sinus invasion, absence of metastasis and longer overall survival. MCT2 expression at the plasma membrane suggests monocarboxylate consumption (likely lactate) from the extracellular environment. Since MCT2 plasma membrane expression is independent from MCT1 or MCT4 (data not shown), MCT2 positive tumors may present a metabolic symbiosis between cancer cells, in which the peripheral and oxygenated oxidative cells consume, through MCT2, the lactate released, through MCT4, by the central and less oxygenated glycolytic cells [34]. As a result, lactate will no longer act in the microenvironment to stimulate tumor aggressiveness. This rationale is in line with the evidence that MCT2 expressing tumors show a less aggressive phenotype than MCT2 negative tumors. Finally, we found CAIX to be more frequently expressed at the plasma membrane of tumors showing high nuclear grade and presence of metastasis, which is in accordance with the contribution of CAIX to the acid-resistant phenotype, rendering cancer cells a survival advantage that will contribute to cancer progression. Also, since CAIX contributes to the extracellular acidification, this pH regulator has a role in the acid-mediated cancer cell invasive behavior [35].

In line with the role of the different proteins in the metabolic remodeling of cancer cells and the evidence from the associations with the clinicopathological data, plasma membrane expression of MCT1 or GLUT1 was identified as a poor prognostic factor and plasma membrane expression of MCT2 was identified as a good prognostic factor for overall survival in univariate analysis. Multivariate analysis showed that none of these markers has a stage-independent prognostic value, differently from results previously described for GLUT1 [21]. Importantly, CD147 cytoplasmic expression was identified as a stage-independent good prognostic factor for overall survival. In disease-free survival, while plasma membrane of GLUT1 was identified as a poor prognostic factor and plasma membrane expression of MCT2 and cytoplasmic expression of CD147 were identified as good prognostic value in univariate analysis, multivariate analysis showed that none of these markers has a stage-independent prognostic value. Importantly, CAIX cytoplasmic expression, while showing borderline significance in the univariate analysis, was identified as a stage-independent good prognostic factor for disease-free survival.

In the present study, we provided evidence for a metabolic reprogramming of adrenocortical malignant tumors towards the hyperglycolytic and acid-resistant phenotype characteristic of the Warburg effect, which associates with a worse prognosis. Since only few studies are available on the metabolic reprogramming of adult adrenocortical carcinoma, we believe that the results from the present study may bring new and relevant knowledge about the biology of this rare type of cancer, with possible developments in adrenocortical carcinoma management and the search for new target-directed therapeutic resources.

## MATERIALS AND METHODS

### Human adrenocortical tumor samples

The series analyzed included 154 formalin-fixed paraffin-embedded adrenocortical neoplasias (76 adenomas and 78 carcinomas, according to Weiss score), retrieved from the files of the Pathology Department of the Clinical Hospital, School of Medicine, University of Sao Paulo, Brazil. No case included in this series fulfills the criteria for oncocytic tumor. Samples were organized into tissue microarrays (TMA) containing 208 cores each (1.0 mm diameter each core). Each case was represented in TMAs by three cores and control samples (kidney) were also included for TMA orientation. Clinicopathological data for the adrenocortical carcinomas included age at diagnosis (normal distribution, mean 40.6 years, range: 15 to 81 years), gender, tumor size (normal distribution, mean 11.8 cm, range: 2.2 to 23.0 cm; categorized using 8 cm as cut-off [36]) and weight (non-normal distribution, median 467.2 mg, range: 10 to 2600 mg), Weiss score and each of its individual histological parameters [37], tumor p53 *status*, staging (according to ENSAT system [38]), metastasis, disease-free survival and overall survival (median 13.5 and 30.5 months, respectively). Cases that could not be evaluated by Weiss score were classified as carcinomas if metastatic disease was detected (clinically malignant). Detailed information of the clinicopathological data for the adrenocortical adenomas as well as the adrenocortical carcinomas is presented in Tables 5 and 6, respectively. The present study was approved by the Local Ethic Committee (number 11090).

**Table 5:** Clinicopathological data of the adrenocortical adenoma patients

Variable	*n*	%
***Age*^#^(*n* = 76)**		
≥ 44.2 years	**35**	46.1
< 44.2 years	**41**	53.9
***Gender* (*n* = 76)**		
Female	**66**	86.8
Male	**10**	13.2
***Tumor size* (*n* = 73)**		
< 8 cm	**71**	97.3
≥ 8 cm	2	2.7
***Tumor weight*^#^ (*n* = 64)**		
< 20.0 mg	**29**	45.3
≥ 20.0 mg	**35**	54.7
***Weiss score* (*n* = 76)**		
0	**35**	46.1
1	**22**	28.9
2	**19**	25.0
Nuclear grade* (*n* = 42)		
Low	**37**	88.1
High	**5**	11.9
Mitotic índex* (*n* = 42)		
Low	**41**	97.6
High	**1**	2.4
***Atypical mitosis* (*n* = 42)**		
Absent	**42**	100.0
Present	**0**	0.0
***Necrosis* (*n* = 42)**		
Absent	**38**	90.5
Present	**4**	9.5
***Venous invasion* (*n* = 42)**		
Absent	**42**	100.0
Present	**0**	0.0
***Sinus invasion* (*n* = 42)**		
Absent	**41**	97.6
Present	**1**	2.4
***Capsular invasion* (*n* = 41)**		
Absent	**38**	92.7
Present	**3**	7.3
***Staging* (*n* = 56)**		
I	**49**	87.5
II	**7**	12.5

# The mean value was used for age cut-off, while the median value was used for weight cut-off as these variables followed a normal and non-normal distribution, respectively.

* Nuclear grade and mitotic index were defined according to Weiss score definitions (30).

**Table 6:** Clinicopathological data of the adrenocortical carcinoma patients

Variable	*n*	%
***Age*^#^ (*n* = 78)**		
≥ 40.6 years	**34**	43.6
< 40.6 years	**44**	56.4
***Gender* (*n* = 78)**		
Female	**61**	78.2
Male	**17**	21.8
***Tumor size* (*n* = 76)**		
< 8 cm	**18**	23.7
≥ 8 cm	**58**	76.3
***Tumor weight*^#^ (*n* = 60)**		
< 467.2 mg	**30**	50.0
≥ 467.2 mg	**30**	50.0
***Weiss score* (*n* = 78)**		
≥ 3^$^	**5**	6.4
3	**13**	16.7
4	**13**	16.7
5	**7**	9.0
6	**11**	14.1
7	**11**	14.1
8	**14**	17.9
9	**4**	5.1
Nuclear grade* (*n* = 41)		
Low	**5**	12.2
High	**36**	87.8
Mitotic índex* (*n* = 41)		
Low	**24**	58.5
High	**17**	41.5
***Atypical mitosis* (*n* = 41)**		
Absent	**28**	68.3
Present	**13**	31.7
***Necrosis* (*n* = 41)**		
Absent	**11**	26.8
Present	**30**	73.2
***Venous invasion* (*n* = 41)**		
Absent	**27**	65.9
Present	**14**	34.1
***Sinus invasion* (*n* = 41)**		
Absent	**35**	85.4
Present	**6**	14.6
***Capsular invasion* (*n* = 40)**		
Absent	**27**	67.5
Present	**13**	32.5
***Staging* (*n* = 76)**		
I	**7**	9.2
II	**36**	47.4
III	**15**	19.7
IV	**18**	23.7
***Metastasis* (*n* = 78)**		
Absent	**37**	47.4
Present	**41**	52.6
***Evolution* (*n* = 78)**		
Alive	**46**	59.0
Dead	**32**	41.0

# The mean value was used for age cut-off, while the median value was used for weight cut-off as these variables followed a normal and non-normal distribution, respectively.

$ Cases classified as carcinoma (Weiss ≥ 3) based on the presence of metastatic disease.

* Nuclear grade and mitotic index were defined according to Weiss score definitions (30).

### Immunohistochemistry

MCT1 immunohistochemistry was performed according to the avidin-biotin-peroxidase complex method (R.T.U. VECTASTAIN Elite ABC Kit (Universal), Vector Laboratories, Burlingame, CA), as previously described [39]. Immunohistochemistry for MCT2, MCT4, GLUT1, CD44 and CAIX was performed according to the streptavidin-biotin-peroxidase complex principle (Ultravision Detection System Anti-polyvalent, HRP, Lab Vision Corporation, Fremont, CA), as previously described [20, 40, 41]. CD147 immunostaining was performed using a polymer system (UltraVision ONE Detection System: HRP Polymer Lab Vision Corporation, Fremont, CA) as already described [42]. Negative controls were performed by the use of appropriate serum controls for the primary antibodies (N1698 and N1699, Dako, Carpinteria, CA). Colon carcinoma tissue was used as positive control for MCT1, MCT4, CD147 and CD44, head and neck squamous cell carcinoma was used for GLUT1, and normal stomach was used for CAIX. Tissue sections were counterstained with hematoxylin and permanently mounted. Please refer to Table 7 for detailed aspects for each antibody used.

**Table 7:** Detailed aspects for each antibody used in immunohistochemistry

Protein	Antigen retrieval	Antibody	Antibody dilution and incubation time
**MCT1**	Citrate buffer (0.01 M, pH = 6), 98°C, 20′	AB3538P Chemicon International	1:200, overnight
**MCT2**	Citrate buffer (0.01 M, pH = 6), 98°C, 20′	sc-50322 Santa Cruz Biotechnology	1:200, 2 hours
**MCT4**	Citrate buffer (0.01 M, pH = 6), 98°C, 20′	sc-50329 Santa Cruz Biotechnology	1:500, 2 hours
**CD147**	EDTA (1 mM, pH = 8), 98°C, 20′	sc-71038 Santa Cruz Biotechnology	1:400, overnight
**CD44**	Citrate buffer (0.01 M, pH = 6), 98°C, 20′	MCA2726 AbD Serotec	1:2000, 2 hours
**GLUT1**	Citrate buffer (0.01 M, pH = 6), 98°C, 20′	ab15309-500 AbCam	1:500, 2 hours
**CAIX**	Citrate buffer (0.01 M, pH = 6), 98°C, 20′	ab15086 AbCam	1:2000, 2 hours

### Immunohistochemical evaluation

Sections were scored semi-quantitatively for expression in cancer cells as follows: 0: no immunoreactive cells; 1: < 5% of immunoreactive cells; 2: 5–50% of immunoreactive cells; and 3: > 50% of immunoreactive cells. Also, intensity of staining was scored semi-qualitatively as follows: 0: negative; 1: weak; 2: intermediate; and 3: strong. The final score was defined as the sum of both parameters (extension and intensity), and, for the metabolism-related proteins, grouped as negative (score 0 and 2) and positive (score 3–6), as previously described [39]. Protein expression in the different cellular localizations (cytoplasm and plasma membrane) was evaluated separately.

### Statistical analysis

Data were stored and analyzed using the IBM SPSS Statistics software (version 20, IBM Company, Armonk, NY). All comparisons were examined for statistical significance using Pearson's chi-square (χ^2^) test and Fisher's exact test (when *n* < 5). Overall survival was defined as the time from the date of primary diagnosis to death related to adrenocortical cancer or last follow-up. Disease-free survival was defined as the time from the date of complete tumor resection to the first radiological evidence of disease relapse or death. Overall and disease-free survival curves were estimated by the method of Kaplan-Meier and data compared using the log-rank test. Predictive factors of prognosis were identified by means of Cox proportional hazards regression models, which were used to estimate hazard ratios (HR) and their 95% confidence intervals in univariate and multivariate analysis. All variables that reached a *p* value < 0.1 at Kaplan-Meier estimates were included in the Cox survival analysis. For multivariate analysis, variables that reached a *p* value < 0.1 at univariate analysis were included. The threshold for significant *p* values was established as *p* < 0.05.
